# An expanding cityscape and its multi-scale effects on lizard distribution

**DOI:** 10.3389/fcosc.2022.839836

**Published:** 2022-07-22

**Authors:** Maria Thaker, Madhura S. Amdekar, Nitya P. Mohanty, Abhijit K. Nageshkumar, Harish Prakash, K. S Seshadri

**Affiliations:** Centre for Ecological Sciences, Indian Institute of Science, Bengaluru, India

**Keywords:** urban - rural gradient, habitat diversity, ALAN, LST (land surface temperature), lizard, drone, GIS - geographic information system, *Psammophilus dorsalis*

## Abstract

Urbanization results in complex and variable changes to environmental conditions, which translate to shifts in selection pressures for organisms. Size of a city as well as the intensity and extent of urbanization can synergistically influence how organisms are impacted. However, less is known about how landscape heterogeneity, rate of land-use change, and scale of urbanization affect species persistence. We evaluate the ways in which urbanization changes the environment and examine how some of these environmental factors influence the presence of the lizard *Psammophilus dorsalis* (Peninsular rock agama), in Bengaluru, India. Variability in environmental factors across the study area was characterised by measures of habitat composition and diversity, habitat connectivity, rate of habitat change, predation pressure, land surface temperature (LST) and artificial light at night (ALAN), that were derived from remotely sensed and citizen science data. Most of these factors showed high variance across two measures of urbanization: distance from city center and proportion of built-up area. Habitat diversity and ALAN were the only two factors that changed predictably and in a non-linear way, with distance from the city center and proportion of built-up area. We then used a multi-scale approach to examine the relative importance of some these environmental factors at the landscape scale, as well as additional factors at the microhabitat-scale, in predicting the presence and relative abundance of *P. dorsalis* respectively. At the landscape scale, LST, which is positively correlated with proportion of cropland, predicted lizard presence; whereas at the microhabitat scale, *P. dorsalis* was more likely to be found in sites with higher proportions of rocks. Overall, we demonstrate that urbanization can result in environmental predictors that do not vary linearly across the urbanization gradient. For the iconic rock agama, many of these environmental factors do not seem to be strong selection pressures that influence their distribution in the expanding cityscape. Whether this urban utilizer can continue to persist with increasing anthropogenic development is uncertain. To better understand drivers of species persistence, we emphasize the importance of quantifying urbanization across multiple axes, considering environmental factors that are relevant to species at different spatial and temporal scales.

## Introduction

As the human footprint on natural ecosystems continues to expand, urbanization has become one of the greatest challenges for biodiversity (McKinney, 2008). Approximately 55% of human population is concentrated in urban areas and this proportion is expected to rise to nearly 68% by the year 2050 (United Nations, 2019). The conversion of natural habitats to cities involves drastic and often permanent changes to the physical structure of environments (Elmqvist et al., 2013; Uchida et al., 2021). For organisms living in these urbanized areas, such structural changes have downstream effects and can influence selection pressures (Sih et al., 2011). For example, the reduction in the diversity of plants in cities negatively impacts insect communities (Vergnes et al., 2014; Sánchez-Bayo and Wyckhuys, 2019), which has consequences for the diet, foraging strategies, and even survival probability of insectivorous birds and reptiles. Even though examples of declining biodiversity in urban areas are now prevalent globally (McKinney, 2008; Huang et al., 2018), what is increasingly being recognised is that the impact of urbanization on organismal responses varies considerably. For instance, species richness of plants can increase in suburban areas, consistent with the intermediate disturbance hypothesis, whereas richness of vertebrate communities often reduces in urbanized areas (McKinney, 2006). Furthermore, the size of a city and intensity of urbanization can synergistically influence how organisms are impacted (McKinney, 2006; Schneider and Woodcock, 2008; Uchida et al., 2021). The term “urban”, therefore, is applied broadly and can obscure the complexity and variation of the underlying ecological processes.

The survival probability and therefore presence of biodiversity in urban areas is directly influenced by how well species respond to the various environmental changes that arise from urbanization. Habitat loss alone accounts for most of biodiversity loss in many cities around the world (Marzluff, 2001; Lim and Sodhi, 2004; Scolozzi and Geneletti, 2012; Paul and Nagendra, 2015; Teitelbaum et al., 2020). Fragmentation or the lack of connectivity of the remaining natural habitats further reduces species richness, especially for species that require large areas for resource acquisition (McKinney, 2008; Vergnes et al., 2014; Beninde et al., 2015) or have low dispersal ability (Stefanescu et al., 2004; Evans et al., 2017). Habitat loss and fragmentation can also result in changes to animal movement patterns (Tucker et al., 2018), habitat use, and distribution (Weller and Ganzhorn, 2004). Given that urbanisation can negatively affect the survival of some species, species composition and community structure in urban areas are different from those in natural ecosystems (Jellinek et al., 2004; Olivier et al., 2020). As species composition changes along urban gradients, predation pressure may also vary (McKinney, 2006; Eötvös et al., 2018; Piontek et al., 2021). Increased predation pressure, especially from feral commensals such as dogs and cats have resulted in significant reductions in small mammal, bird, and reptile populations in urban areas (Iverson, 1978; Baker et al., 2005; Sudhira and Nagendra, 2013; French et al., 2018). Reduced or no change in predation pressure in some cities are also common (Balakrishna et al., 2021), and this can increase prey survival probability, but also increase intra-specific or inter-specific competition within the fragmented urban habitat. Among the abiotic factors that change with urbanization, temperature and artificial light are the most pervasive. Land Surface Temperature (LST) is typically higher in urban areas, resulting in what is known as the urban heat island effect (Peng et al., 2012; Lee et al., 2014). Several studies associate the reduction of vegetation and increase in LST as an immediate effect of urbanization (Nalini, 2021; Yamak et al., 2021). An increase in LST could, however, be beneficial to some species; for example, the increase in grey-headed flying foxes *Pteropus poliocephalus* that roost in Melbourne has been attributed to higher urban temperatures (Parris and Hazell, 2005). The ecological effects of artificial light at night (ALAN) are abundant (Frank et al., 2006; Gaston et al., 2013) and include changes to animal behaviour, physiology and reproduction (Newport et al., 2014), as well as species interactions (Knop et al., 2017). Overall, these numerous changes to environmental and ecological factors result in changes to species responses which in turn can affect biodiversity in urban environments.

Here, we quantify the ways in which urbanization changes the environment and examine how these potential environmental factors in turn influence the presence of the Peninsular rock agama (*Psammophilus dorsalis*) in one of the fastest growing cities in India. Among the reptile species found in the city of Bengaluru, *P. dorsalis*) is the most prominent lizard. This species is found across southern India, in semi-arid areas that are typically characterised by rocky habitat with boulders and sheet rocks interspersed with scrub vegetation (Radder et al., 2005). Despite considerable evidence of this species’ ability to be an urban utilizer (Amdekar et al., 2018; Batabyal and Thaker, 2019b), what is unclear is whether environmental factors influence their distribution and abundance in urban areas. For example, habitat composition, including vegetation cover is an important factor that influences insect diversity and density, the primary source of food for the insectivorous *P. dorsalis* (Balakrishna et al., 2016). The availability of rocks and boulders, or artificial substrates with similar structural properties, are used as active and sleep sites, and therefore, are also critical resources for this agamid lizard (Radder et al., 2005; Mohanty et al., 2021). Similarly, higher LST could be beneficial for efficient thermoregulation, whereas ALAN would be disruptive for sleep (Aulsebrook et al., 2018). Therefore, the presence and relative abundance of *P. dorsalis* is likely to be determined by processes operating at both the landscape and microhabitat scales.

We started by characterizing urbanization in Bengaluru, based on a range of environmental factors at the landscape scale. These factors include habitat composition, habitat connectivity and diversity, rate of habitat change, avian predation pressure, LST and ALAN, which were derived from remote sensed and citizen science data. We then examined how different landcover types and environmental factors change across two measures of urbanization: ‘distance from the city centre’, which captures the somewhat concentric pattern of urban expansion in Bengaluru, and ‘proportion of built-up’, which directly captures urbanization. We predict that these two measures of urbanization will be tightly correlated and that habitat connectivity, habitat diversity, rate of habitat change, and avian predation pressure would decrease with increasing degree of urbanization, whereas LST and ALAN would increase with urbanization. In addition to the landscape-level information, we captured microhabitat level data, including microhabitat composition and vegetation height, using targeted drone imagery and on-ground measurements in a smaller subset of locations across the study area. These data enabled us to examine the form and pattern of urbanization and to use a multi-scale approach to predict the presence of the agamid lizard species at the landscape scale and their abundance at the microhabitat scale. We predict that these various measures of urbanization will negatively impact both presence and abundance of the lizard in a rapidly growing metropolis in India.

## Materials and methods

### Study area

Bengaluru (12.59° N, 77.57° E), the administrative capital of the Karnataka State in southern India, is one of the fastest growing cities in India (Sudhira et al., 2007). The city is known to have been in existence since the 9^th^ century, likely as a small village, which by the 19^th^ century, turned into a prominent centre for trade and commerce (Sudhira et al., 2007). Bengaluru receives a mean annual rainfall of about 880 mm and annual temperatures range between 12-38°C. The landscape in and around this city is undulating; the region is part of the geologically ancient peninsular gneissic complex comprising the Dharwar craton and is located on the Deccan plateau at an elevation of ca. 900 m (Valdiya, 2001). Monolithic formations of granite and rolling hillocks of peninsular gneiss are a characteristic feature of the landscape, running north to south through Bengaluru, forming ridges which delineate three watersheds within the city (Sudhira et al., 2007).

Currently, 12.34 million people are estimated to live in Bengaluru and this number is expected to increase by nearly 32% over the next decade (United Nations, 2019). The rapid development and urban sprawl of Bengaluru over the last few decades (Sudhira and Nagendra, 2013) has resulted in the city encompassing an estimated area of 1219.5 km^2^ spanning 251 villages (Bangalore Development Authority, 2017). Expectedly, the oldest and most urbanized areas of the city are densely populated and almost completely dominated by anthropogenic structures (Shetty et al., 2012). Rate of urbanization, however, is reduced in the center of the city owing to scarcity of land and high land prices (Sudhira et al., 2003) but is accelerated and fragmented towards the periphery (Schneider and Woodcock, 2008; Taubenböck et al., 2009). Correspondingly, rate of vegetation loss and habitat fragmentation are stable within the core but are increasing rapidly in the periphery (Jaganmohan et al., 2012). Despite widespread urbanization, vegetation, such as trees and scrub habitats, persist around water bodies and in home gardens, parks, educational institutes, and remnant forests (Sudha and Ravindranath, 2000; Nagendra and Gopal, 2011; Jaganmohan et al., 2012; Gopal et al., 2015). Approximately 38 species of reptiles have been documented in Bangalore within a 40 km radius from the city (Karthikeyan, 1999) and the matrix of urban parks and large green spaces sustain a rich diversity of potential predators of reptiles (e.g., birds) and prey, such as insects (Kumar et al., 1997; Savitha et al., 2008).

### *Psammophilus dorsalis* in Bengaluru

In the urban areas of Bengaluru, *P. dorsalis* show key changes to its behaviour, morphology, and physiology that reflect its flexibility and adaptation to novel anthropogenic conditions. In the foraging context, urban lizards show a shift in diet composition and foraging strategies (Balakrishna et al., 2016). Lizards in the urban areas are also less risk averse, as measured by their shorter flight initiation distances from an approaching human (Batabyal et al., 2017), and are faster to learn the locations of safe refuges (Batabyal and Thaker, 2019a). The behavioural flexibility of this species extends to the selection of sleep sites that limit artificial illumination in urban areas (Mohanty et al., 2021). In the social and sexual context, males of *P. dorsalis* utilise a reactive social coping style in urban areas, unlike the proactive strategy of males in rural areas (Batabyal et al., 2017; Batabyal and Thaker, 2019b). Morphological differences in body size and limb dimensions between urban and rural populations (Balakrishna et al., 2021) seem to suggest adaptations to effectively utilise human-made substrates and environments. Despite all these changes, *P. dorsalis* in the city show almost no negative health indicators as measured by body condition, ectoparasite load, and cell mediated immune response of individuals (Amdekar et al., 2018; Batabyal and Thaker, 2019b).

### Environmental factors and urbanization

**Figure 1:**
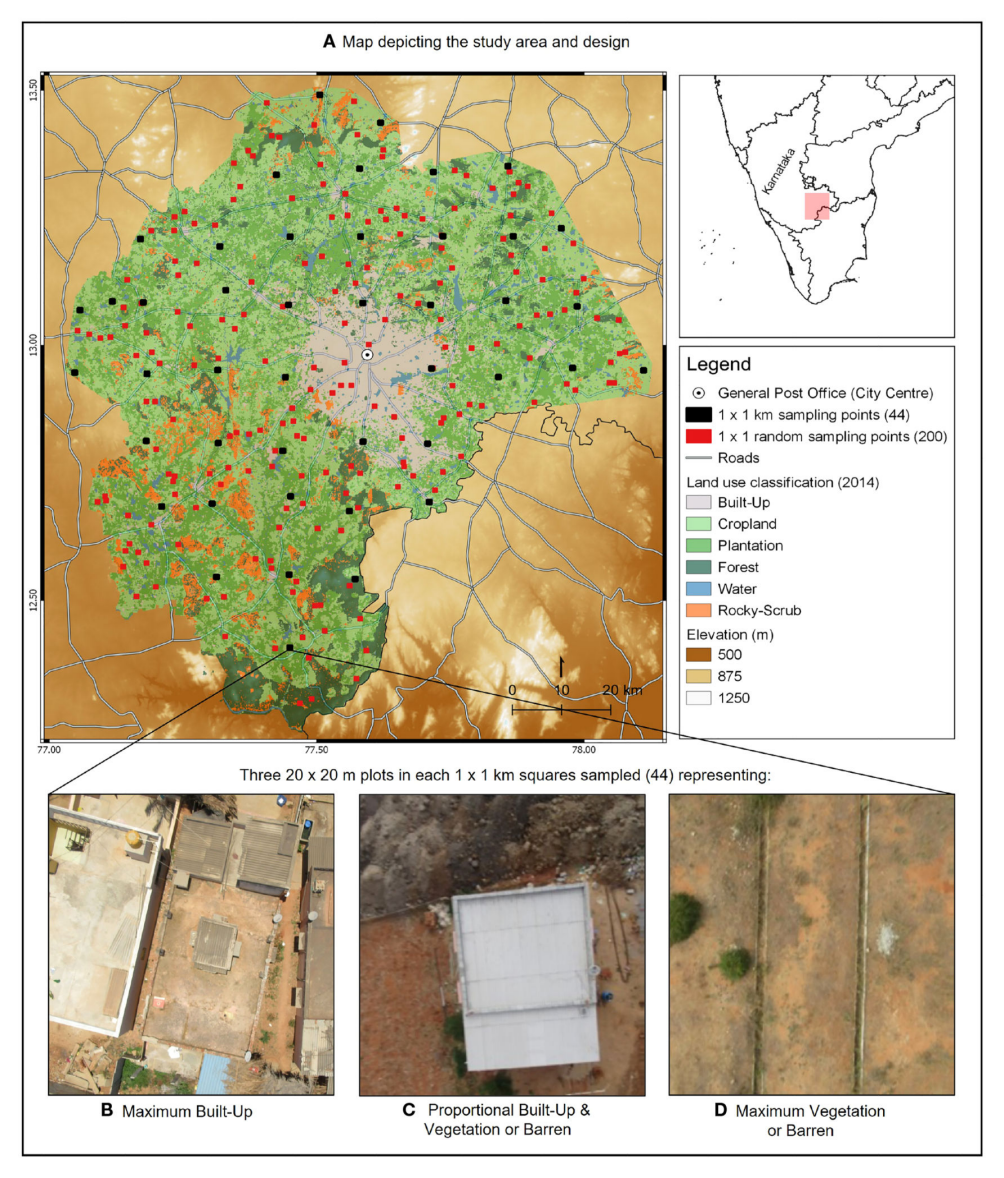
**(A)** Environmental factors associated with urbanization in Bengaluru, Karnataka State, India, were characterised at 200 locations of 1 km^2^ (randomly placed red points on map). To determine the presence and abundance of *Psammophilus dorsalis*, 44 locations of 1 km^2^ were sampled (evenly spaced black squares on map). In each sampling location, three plots of 20 m^2^ each were chosen to represent the following: **(B)** maximum built-up, **(C)** proportional built-up and vegetation/barren and **(D)** maximum barren or vegetation (shown are representative drone images). Microhabitat factors and presence and abundance of *P. dorsalis* were recorded in each of these plots.

To evaluate the ways in which environmental variables change with urbanization, we delineated a study area of 9255 km^2^, starting from the centre of the city, landmarked by the general post office, and extending at most to 82 km (Figure 1). Within this study area, we overlaid 200 random points, measuring 1 km^2^, spaced at least 0.5 km apart and extracted information to calculate LST, ALAN, predation pressure, habitat contiguity, habitat diversity, and rate of habitat change.

Thermal environment, characterised from the daytime land surface temperature obtained from the MOD11A1 data of the Moderate Resolution Imaging Spectroradiometer (Wan, 2008) were used to generate LST (in °Celsius) for as many days in the months of May and June 2016 that data was available. This period coincides with the peak of summer in the region and overlaps with the lizard sampling period described below. ALAN values were obtained for 2013 (the last available year) from Version 4 DMSP-OLS Night-time Lights Time Series data (Baugh et al., 2010). Predation pressure was calculated as an Avian predator index using data available on ‘eBird’ database (Sullivan et al., 2009). For this, we listed 48 species as potential predators of *P. dorsalis* based on published literature (Ali and Ripley, 1983), photographs (eBird, 2021; India Nature Watch, 2021), as well as personal observations (HP and KS). The observation records of the potential predators were filtered for the study area between years 2015 and 2017 to only include observations from stationary counts. To account for spatial and temporal autocorrelation in the data, we randomly selected only one sampling event within 10x10 km grids per week, which resulted in 4131 counts of bird occurrences. From this subsampled data, the observed bird counts were spatially interpolated using a triangulated irregular networks (TIN) method in QGIS (version 3.10.12-A Coruña), which creates an interpolated surface formed by triangles connecting the nearest neighbour. The bird count points were used to generate values for each grid at a 10.7 x 10.5 km scale.

Land use variables were computed from remotely sensed landcover imagery from the ‘Bhuvan’ repository (LULC 250k, National Remote Sensing Centre, ISRO, Government of India, Hyderabad, India). Data from the year 2014-2015 was used for contemporary land cover and historical change was computed as the difference between years 2005-2006 and 2014-2015. These time points were chosen because 2005-2006 was the earliest and 2014-2015 was the latest error-free imagery available on the Bhuvan repository and enabled us to calculate landcover change over a 9-year period. The pre-classified imagery was re-classified to 6 focal classes (Built-Up, Cropland, Rocky-scrub, Plantation, Water, Forest). Habitat diversity was calculated using Simpson’s Diversity Index, computed as 1 minus the sum, across all patch types, of the proportional abundance of each patch type, squared. Values of the diversity index ranged from 0-1 wherein 0 denoted a single landcover type and 1 denoted the maximum number and configuration of landcover types. Habitat contiguity was calculated for ‘rocky-scrub’ since this land-class is likely to be the most relevant habitat of the lizard. Contiguity was measured as the percentage of “like adjacency” by counting the number of adjoining pixels of rocky scrub land use class and dividing it by the number of pixels adjoining rocky scrub and other land use classes. The resulting value was multiplied by 100 to convert it into a percentage. Both habitat diversity and contiguity were computed using Fragstats V4.2 (McGarigal and Marks, 1995). Finally, rate of habitat change was calculated only for the rocky-scrub land-use class as the difference in the proportion of this land cover between the years 2014-15 and 2005-06. Negative values indicate greater conversion of rocky-scrub to other habitat types over this time period.

### Sampling for lizards

Lizards were sampled at two scales using a nested design across the study area (Figure 1). During May and June 2016, we surveyed 44 areas (thereafter, square) of 1 km^2^ that were located uniformly across the study area and at least 15 km apart. Within each square, we visually assessed and chose three plots of 20 x 20 m where we determined the presence and relative abundance of *P. dorsalis*. To capture the widest range of microhabitat variation, these selected plots differed in habitat composition, such that one plot was primarily built-up with human-made structures, one was approximately equal proportions of built-up and vegetation/barren land, and one was primarily vegetation/barren land, wherever possible. Some plots (n = 9) were missing or excluded because the habitat composition criteria above were not met, or if there was some interference preventing us from the survey. This resulted in 123 plots and within each plot, a visual encounter survey of 30 min. duration was carried out by two persons. We recorded the sex and number of individuals of *P. dorsalis* encountered. If we failed to detect any lizard in all three plots, we scored the 1 x 1 km square as ‘lizard absent’ and if lizards were detected in at least one plot, the square was marked as ‘lizard present’ for analysis. We used the presence of lizards at the 1x1 km square to determine landscape-level predictors and the abundance of lizards at the 20 x 20 m plot to determine microhabitat-level predictors.

### Environmental predictors of lizards at the landscape and microhabitat scales

Landscape scale environmental predictors of lizard presence at the 1x1 km square scale (n = 44) was obtained using the same steps mentioned in section 3. To capture the potential predictors of lizard abundance at the microhabitat scale, we quantified fine scale proportions of land cover types in each of the 20 m^2^ plots (n = 123) using images captured from a custom-built drone (Sree Sai Aerotech) equipped with a Wi-Fi enabled camera (Generic Colour Camera, 48 megapixels). We flew the drone between 30 – 80 m and captured at least 5 images at each lizard sampling site (Figure 1). The raw images were visually sorted to select the best image that encompassed the 20 m^2^ sampling area and were classified based on pixel values using an ‘unsupervised classification method’ in ArcGIS (10.2) with up to 5 additional rounds of supervised classification and manual corrections. The images were classified as vegetation (includes naturally growing and cultivated grass, crops and shrubbery), bare soil, rocks (includes small rocks, boulders, and sheet rocks), and construction. We also estimated vegetation height (m) in each plot as an average height of all plants.

### Data analysis

To examine the change in environmental variables across degrees of urbanization, we ran two sets of models that determined the relationship between LST, ALAN, avian predation index, habitat contiguity of rocky-scrub, Simpson’s habitat diversity index, and rate of change of rocky-scrub with (1) distance to the city centre and (2) proportion of built-up as separate predictors. For these, we used generalised additive models (GAM) with gaussian family (link: identity) as error distribution to detect non-linear trends, if any (in R, version 4.1.0 and package MGVC (Mixed GAM Computation Vehicle with Automatic Smoothness Estimation)). GAM models were also compared with their linear counterparts to determine which model better explained the deviance. We also examined the relationships between distance from the city centre and the proportions of all landcover classes, including the built-up class using GAM, with gaussian family as described above, and with linear models for comparison.

To determine the key predictors of *P. dorsalis* occurrence at the scale of 1 x 1 km (n = 44), we built seven candidate models comprising a global model, models with single predictors, and a null model based on *a priori* chosen predictors – LST, avian predation index, proportions of built-up and rocky-scrub landcover types, and rate of change (rocky-scrub). We did not include ALAN, habitat contiguity (rocky-scrub), habitat diversity, proportion of cropland, and distance from city centre due to collinearity with other predictors (see Pearson’s correlation in Supplementary Table 1) and to avoid overparameterizing the global model. We scaled all the predictors by their mean to make them comparable and built generalized linear models with binomial error distributions. Models were selected based on Akaike Information Criterion values (Burnham and Anderson, 2002). We generated adjusted R^2^ values only for the models that were statistically significant using the *rsq* package.

At the microhabitat (20 x 20 m) scale, we ran a zero inflated generalised linear mixed effect model to determine the microhabitat predictors influencing the observed abundance of *P. dorsalis*. Abundance of the lizard was used as a response variable with the following predictors – distance from the city centre, proportion of rocks, vegetation height, proportion of construction, and proportion of bare soil (after taking collinearity of the predictors into account). We used a negative binomial distribution as the error family and incorporated zero inflation to account for the large number of absences (0s) in the response variable. The square ID was used as a random effect to account for any potential spatial bias across the 1 x 1 km sampling locations. The model was run using the package ‘glmmTMB’ in R (Brooks et al., 2017).

## Results

### Change in environmental variables across two measures of urbanization

**Figure 2:**
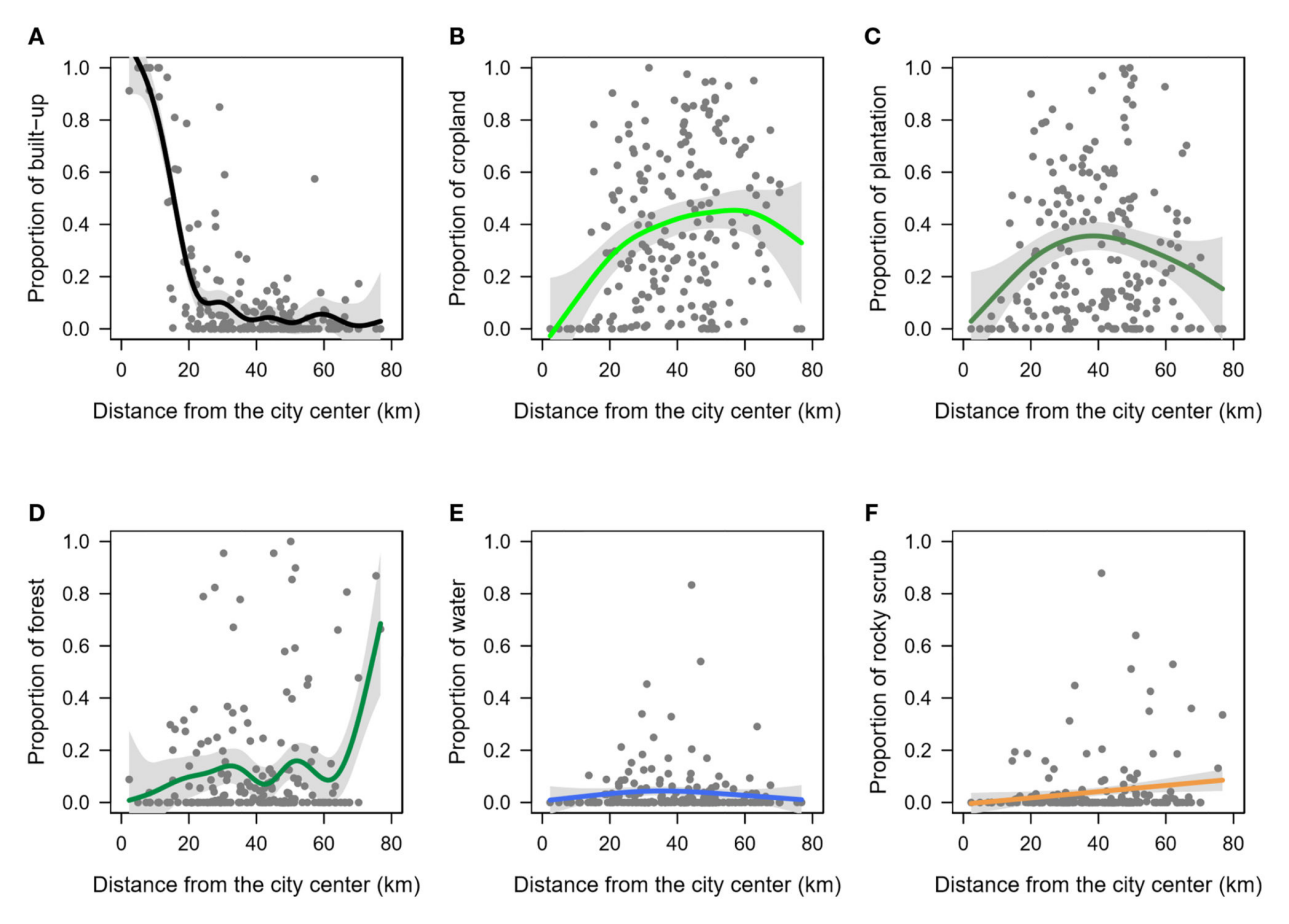
Relationship between the proportions of each landcover type and the distance from city centre. Gray shaded region indicates the confidence intervals along the trendline. **(A)** built-up, **(B)** cropland, **(C)** plantation, **(D)** forest, **(E)** water, and **(F)** rocky scrub.

Variation in the proportion of land cover types across distance from city centre were better explained by the GAMs than the linear models (Supplementary Table 2), and hence, only the results from the non-linear models are described here. As expected, the two measures of urbanization were non-linear and strongly correlated, such that the proportion of built-up steadily declined until it remained low across distances from approximately 20 km to 80 km from the city centre (p-value <0.01, deviance explained=70.4%, Figure 2A, Supplementary Table 2). The other land use classes such as crop, plantation, forest, water and rocky scrub were found in greater proportions >20 km away from the city centre (Figures 2B–F) but showed high variance and a weak correlation with distance from city centre (deviance explained ≤12%, Supplementary Table 2).

**Figure 3:**
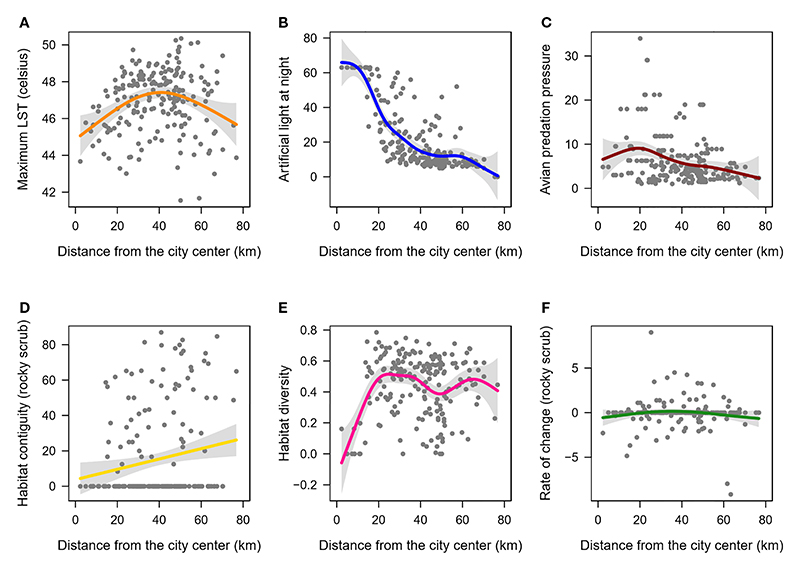
Patterns of change in environmental factors as a function of distance from the city centre. Shown are the non-linear relationships (solid-coloured lines) of **(A)** LST, **(B)** ALAN **(C)** Avian predation pressure, **(D)** Habitat contiguity for the rocky-scrub landcover **(E)** Habitat diversity, and **(F)** Rate of change for the rocky-scrub landcover. Gray shaded region indicates the confidence intervals along the trendline.

Environmental factors showed high variance across the two measures of urbanization, distance from the city centre (Figure 3) and proportion of built-up (Supplementary Table 3, Supplementary Figure 1). Non-linear models (GAMs) were better fits to the data than linear models (Supplementary Table 3). Specifically, ALAN decreased non-linearly with distance from the city centre (smooth term p-value <0.01, deviance explained=64.6%, Figure 3B) and increased non-linearly with proportion of built-up (smooth term p-value <0.01, deviance explained=65%, Supplementary Figure 1B). Habitat diversity index increased initially with distance from the city centre and then appeared to flatten (smooth term p-value <0.01, deviance explained=20.5%, Figure 3E). Habitat diversity also decreased non-linearly with increase in proportion of built-up (smooth term p-value <0.01, deviance explained=29.4%) and distance from city centre (smooth term p-value <0.01, deviance explained=20.5%). The remaining four response environmental factors – LST, avian predation pressure, habitat contiguity (rocky-scrub), and rate of change (rocky-scrub), explained ≤12% of the deviance with distance from the city centre and proportion of built-up (Figure 3, Supplementary Figure 1, Supplementary Table 3).

### Predictors of *P. dorsalis* presence at the landscape scale

**Figure 4:**
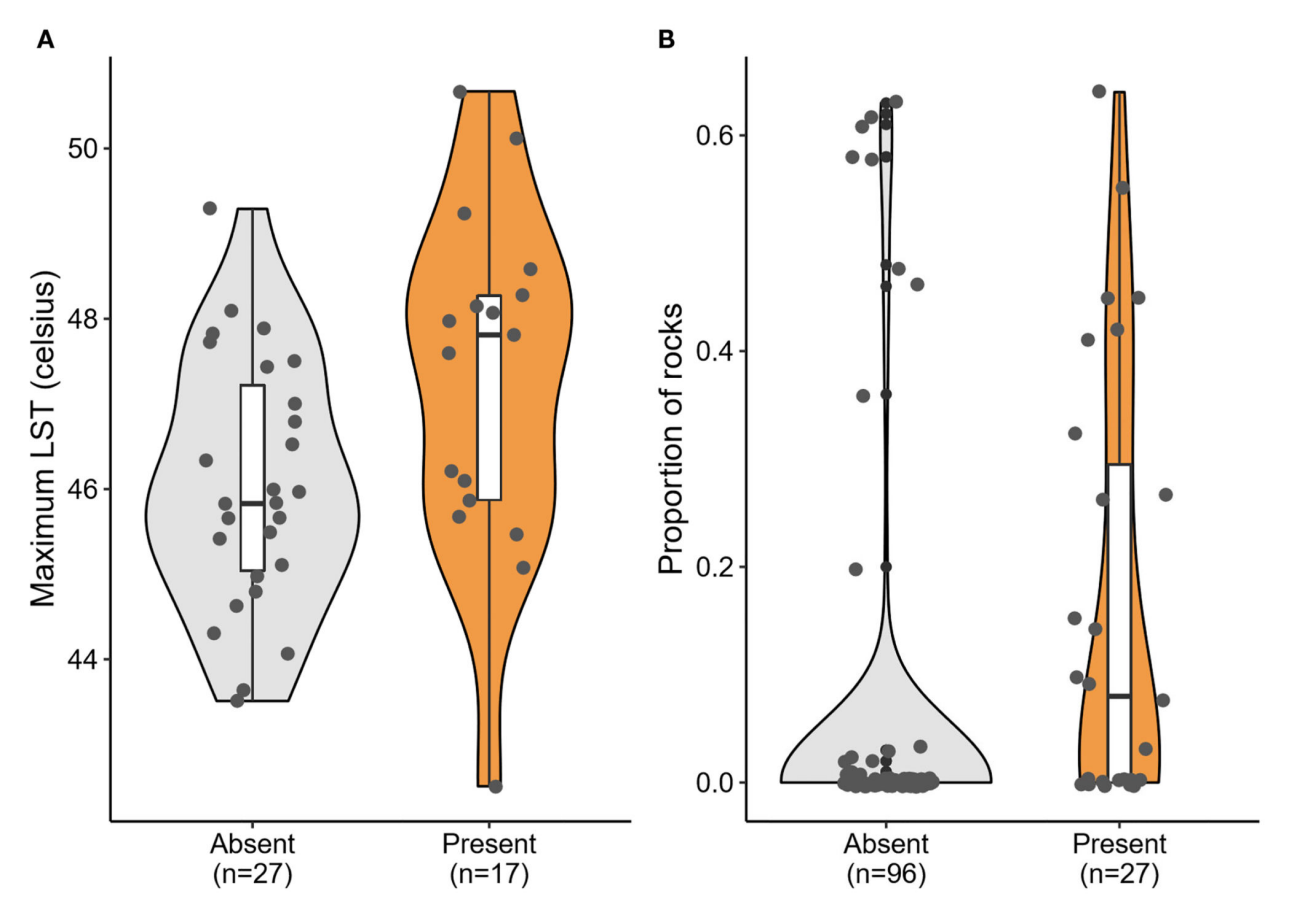
Significant predictors of *Psammophilus dorsalis* presence at the **(A)** landscape scale (1 x 1 km square) and **(B)** the fine scale (20 x 20 m plots), in and around Bengaluru city, India. Box plots within violins display median, quartiles, 5^th^ and 95^th^ percentiles and extreme values.

Of the seven candidate models built to predict the presence of *P. dorsalis* at the landscape scale (1 x 1 km square), a single-predictor model with LST performed the best (Table 1, Figure 4A). Lizard presence was positively associated with LST (*β* = 0.76, SE = 0.36, *p* = 0.036). However, this model explained only 10.07% of the variation in lizard presence, and all other models were as weak as the null model (Table 1). LST is also positively correlated with the proportion of cropland (r = 0.54, p < 0.001; see Supplementary Table 1).

### Predictors of *P. dorsalis* abundance at the microhabitat scale

At the microhabitat scale (20 x 20 m plot), none of the environmental predictors significantly explained the abundance of the lizards (see conditional model in Table 2). However, the association between proportion of rocks and the probability of lizard absence was statistically significant in the zero-inflation model (*β* = -4.51; *p* = 0.006; Table 2), i.e., sites with rocks had fewer absences of *P. dorsalis* than sites without rocks (Table 2, Figure 4B).

## Discussion

Urbanization alters several biotic and abiotic components of an ecosystem. The patterns of change in these components greatly differ from one city to another, depending on various factors, including the size of the city, magnitude of change, and form of urban development (Uchida et al., 2021). Our study examines the magnitude and direction of change in different environmental factors in response to two measures of urbanization in Bengaluru, distance from city center, which reflects the mode of urban expansion, and proportion of built-up, which captures the degree of urbanization. We find that the relationship of only two factors, ALAN and habitat diversity, scaled in predictable but non-linear ways with these measures of urbanization. All other factors, such as LST, avian predation pressure, habitat contiguity (rocky-scrub), and rate of change (rocky-scrub) were highly variable and showed no consistent scaling across the extent of urbanization. For the iconic Peninsular rock agama, *P. dorsalis*, LST at the landscape scale and the proportion of rocky substrate at the microhabitat scale predicted their distribution in the city. Thus, although urbanization results in a range of human-induced rapid environmental change (Sih et al., 2011), many of these potential selection pressures may not directly influence species presence at relatively large or fine scales.

Our examination of environmental factors across varying degrees of urbanization in Bengaluru revealed several interesting patterns that reflect high heterogeneity and non-uniform scaling. Given the rapid expansion in the field of urban ecology in the last few decades, it is not surprising that measures of ‘urbanization’ vary between studies (Szulkin et al., 2020). For example, many studies consider the distance from the center of the city as a measure of urbanization while others use anthropogenic development or human population density. Different measures, however, capture different aspects of urbanization and can influence the interpretation of the impacts on ecosystems and biodiversity (Szulkin et al., 2020). Despite the strong correlation between ‘distance from the city center’ and ‘proportion of built-up’ for Bengaluru, we find that most ecological factors showed no discernable patterns or associations with either of these measures of urbanization.

Many studies have shown an ‘urban heat island effect’ as a common outcome of urbanization (Battles and Kolbe, 2019; Yamak et al., 2021). However, we find no evidence of elevated temperatures (LST) in the urban core or where anthropogenic built-up areas were highest. Lack of a positive relationship between urbanization and LST can be attributed to Bengaluru’s high altitude (~900m) location on the Deccan plateau and the relatively dense vegetation across the city (Siddiqui et al., 2021). As expected, ALAN was extremely high in the urbanized areas and this anthropogenic alteration to the environment rapidly reduced away from city center. Notably, intensity of ALAN was highly variable in the rural areas, where the ‘built-up’ landcover class was relatively low (ranging from 0-25%). For taxa that are negatively affected by ALAN, rural areas with high levels of night light (e.g., some villages, cropfields or plantations) may pose a challenge. We also found higher densities of avian predators closer to the city center as opposed to rural areas, suggesting that urbanized areas provide avian predators with sufficient refuge and prey to sustain abundant populations (McCabe et al., 2018). This pattern of higher avian predators in urban areas has also been documented in other Indian cities, such as New Delhi where raptors are subsidized by active feeding and the unrestricted access to food waste and their associated prey (Kumar et al., 2018). In addition to avian predators, feral dogs are known predators of *P. dorsalis* (Amdekar and Thaker, 2019), and although cities across India have relatively high populations of feral dogs (Home et al., 2018), information on the relative abundance and distribution patterns of dogs in Bengaluru is lacking. Thus, the spatial distribution of predation pressure for lizards may be incomplete.

The ongoing process of urbanization means that modifications to existing habitat structure can occur at different times and rates. Drastic changes to habitats seldom occur in highly urbanized areas due to the already saturated constructed spaces, whereas rapid changes may be evident in areas transitioning from rural to suburban spaces. In our study area, we find that the most urbanized area in Bengaluru is characterized by a highly developed ‘built-up’ core within 20 km of the city center. This core is surrounded by a matrix of cropland, plantations, rocky-scrub, and water bodies. The ‘rocky-scrub’ land cover class is among the most susceptible to anthropogenic alteration, but contrary to our expectation, the rate of change in this landcover type varied across our study site with no clear urban to rural gradient. In areas where a change in the proportion of habitat types was observed, the rocky-scrub habitat appeared to be converted to other land use classes, such as croplands or plantations, and to a lesser extent, into built-ups areas. Urbanization is typically characterized by a replacement of natural vegetation and terrain with artificial structures, leading to reduced habitat diversity and contiguity (McKinney, 2006; Uchida et al., 2021). Such changes to natural habitats were apparent in our study, as less urbanized areas showed higher habitat diversity and greater habitat contiguity. Despite the overall patterns of urban-induced change in the environmental factors that we quantified here, what was most apparent was the high variation across the entire study area. As others have indicated, urbanization develops non-uniformly, resulting in heterogenous patterns of important environmental drivers across the urban-rural gradient (McDonnell et al., 2012; Uchida et al., 2021). For example, a reduction of natural habitat contiguity combined with increasing LST could negatively impact insect abundance, which in turn can have bottom-up consequences for their predators. Landscape heterogeneity, therefore has the potential to result in emergent properties for the ecosystem, by disproportionately increasing the risk for some species, or the ecosystem, when particular environmental drivers interact (Newman et al., 2019).

For many species, urbanization increases selection pressures, but for others, urban-induced changes to the environment can relax the pressure. Relative importance of these selection pressures for species, however, may depend on their spatial scale. At the landscape scale, we find that LST predicted lizard presence across the city. As intuitively appealing as it is to attribute higher surface temperatures as a positive effect for an ectothermic species, such as *P. dorsalis* (Diamond et al., 2018; Diamond and Martin, 2020), this predictor explained limited variation in lizard presence at the landscape scale. LST is also positively correlated with the proportion of cropland, which suggests that these drivers may interact to influence the suitability of the habitat for lizards. In this region, agriculture is a mosaic of crops across small land holdings, and this heterogeneity of crops could support high insect diversity and availability within and around the cropland matrix (Sirami et al., 2019), thereby providing insect prey for lizards (Balakrishna et al., 2016). Alterations at the microhabitat scale may affect animal survival and distribution in a different way from those at the landscape scale. At the microhabitat scale, *P. dorsalis* was more likely to be found in sites with higher proportions of rocks, which is consistent with the general microhabitat preferences and geographic distribution for the species (Radder et al., 2005). None of the other large-scale or fine-scaled predictors adequately explained lizard abundance, suggesting that the environmental variation that we find at both spatial scales only weakly predict lizard presence, regardless of lizard density.

Effects of urbanization on the distribution and abundance of lizards in other studies are similarly inconsistent (French et al., 2018). Several lizards, such as *Lacerta agilis*, readily occupy urbanized and natural habitats (Becker and Buchholz, 2016), whereas other species, such as *Anolis cristatellus* and *Plestiodon reynoldsi* have been found to prefer natural habitats, with the latter being absent from urbanized areas altogether (Pike and Roznik, 2009; Kolbe et al., 2016; Battles and Kolbe, 2019). Many have recognized that heterogeneity in the way cities develop and the resulting mosaic of natural and modified habitats may better represent urban-induced environment change (McDonnell et al., 2012; Ramalho and Hobbs, 2012; Szulkin et al., 2020). Our approach to capture this heterogeneity was to calculate a habitat diversity index and a habitat connectivity index, but these measures showed extremely high spatial variation. Thus, like in many developing cities, the pattern of urbanization in Bengaluru is spatially variable (McDonnell et al., 2012; Szulkin et al., 2020). Temporal variation in anthropogenic development is an additional feature of urbanization that captures environmental stability (Ramalho and Hobbs, 2012). This is particularly relevant for species that are territorial, and are consequently exposed to habitat development within their dispersal range over their lifetime. Temporal dynamics of landscape change, measured as the rate of rocky-scrub conversion over a nine year period, did not seem to influence lizard distribution across the city. In spatio-temporally dynamic cities such as Bengaluru, the presence of *P. dorsalis* may depend on several factors that we could not directly quantify, such as the availability of insect prey and microhabitat-scale thermal heterogeneity.

Overall, our multiscale sampling approach and use of remote sensing data from both drone imagery and satellite sensors, allowed us to generate several insights about urbanization and its effects on lizards. Not surprisingly, we find that spatial and temporal changes to the environment caused by urbanization are highly variable and non-linear, and thus, urban-rural ‘gradients’ are difficult to characterize and generalize. The fact that environmental factors scale in different ways depending on whether urban intensity is measured as the distance from the city center or as the proportion of anthropogenic built-up reinforces the challenge in defining urbanization. The ways in which the environmental effects of urbanization scale across space can influence the relative importance of these potential selection factors for species survival (Uchida et al., 2021). For an urban utilizer like *P. dorsalis*, which is known to exhibit high behavioral and physiological flexibility in response to urbanization (Amdekar et al., 2018; Batabyal and Thaker, 2019a; Mohanty et al., 2021), most features of urbanization showed little effect on its distribution and abundance. However, not all species are expected to respond as urban utilizers (Fischer et al., 2015), and studies that increase spatial and temporal resolution of sampling will be needed to understand the distribution patterns of those taxa and of biodiversity in general. Whether *P. dorsalis* can sustain continual habitat and environmental changes during the urbanization process remains uncertain. We emphasize the need to examine urbanization as a suite of shifted selection pressures that are appropriately scaled to the taxa of interest (Szulkin et al., 2020), as a way to understand persistence and distribution in this novel and now ubiquitous habitat.

## Supplementary Material

Supplementary Material The Supplementary Material for this article can be found online at: https://www.frontiersin.org/articles/10.3389/fcosc.2022.839836/full#supplementary-material

## Figures and Tables

**Table 1:** Generalized linear models predicting the presence of *Psammophilus dorsalis* in the 1 km x 1 km squares (n = 44), in and around Bengaluru city, India.

Parameters	k	ΔAIC	weight
LST	2	0	0.56
Null	1	3.11	0.12
RoC (Rocky-scrub)	2	3.45	0.10
Predator	2	3.85	0.08
Rocky-scrub	2	4.44	0.06
Built-up	2	4.56	0.06
Built-up + Rocky-scrub + RoC (Rocky-scrub) + LST + Predator	5	6.05	0.03

Predictors include land surface temperature (LST), avian predation pressure (Predator), proportions of built-up and rocky-scrub landcover type (current) and rate of change (RoC) of rocky-scrub (between 2005-06 to 2014-15).

ΔAIC is the difference in Akaike information criterion values (AIC) with the best model; weight (Akaike weight) is the relative support a model has from the data compared to the other models in the set.

**Table 2:** *Zero inflated* generalized linear mixed models to predict the abundance of *Psammophilus dorsalis* at the microhabitat scale (20 x 20 m plots) in and around Bengaluru City, India.

	*Conditional model*					*Zero inflation model*			
	Estimate	Std. Error	z value	Pr(>|z|)		Estimate	Std. Error	z value	Pr(>|z|)
(Intercept)	1.067	0.969	1.100	0.271		3.956	1.305	3.030	0.002
Proportion of rocks	0.620	0.893	0.694	0.488		-4.515	1.639	-2.755	0.006
Average vegetation height	-0.003	0.003	-0.780	0.435		-0.012	0.021	-0.591	0.555
Distance from city centre	0.007	0.018	0.399	0.690		-0.008	0.007	-1.144	0.253
Proportion of construction	-0.882	1.067	-0.827	0.408		-2.686	1.474	-1.822	0.068
Proportion of bare soil	-0.328	0.890	-0.369	0.712		-2.006	1.403	-1.430	0.153

The conditional model estimates explain the abundance of the lizard and the zero-inflation of the model estimates explain the absence of the lizard in the site.

## Data Availability

The raw data supporting the conclusions of this article will be made available by the authors, without undue reservation.
