# Supplementary material for: An expanding cityscape and its multi-scale effects on lizard distribution
Source: Front Conserv Sci. Author manuscript; Available in PMC 2024 Mar 29. (PMC7615779; doi:10.3389/fcosc.2022.839836)

**SUPPLEMENTARY INFORMATION**

**Manuscript Title:**

An expanding cityscape and its multi-scale effects on lizard distribution

# Authors:

# Maria Thaker^†^, Madhura S. Amdekar, Nitya P. Mohanty, Abhijit K. Nageshkumar, Harish Prakash, K. S. Seshadri

**Affiliation:**

Centre for Ecological Sciences, Indian Institute of Science, Bengaluru 560092, India

^†^ Apart from the first author, all other authors have been listed in alphabetical order

# Correspondence:

Maria Thaker: [mthaker@iisc.ac.in](mailto:mthaker@iisc.ac.in); Centre for Ecological Sciences, Indian Institute of Science, Bengaluru 560092, India

**Supplementary Table 1.** Pearson’s correlation coefficient and the corresponding p-values in parentheses for the environmental predictors of lizard presence at the landscape level.

|  | **LST** | **ALAN** | **Predation pressure** | **Distance from city center** | **Proportion of built-up** | **Proportion of**  **rocky scrub** | **Rate of change**  **(rocky scrub)** | **Habitat contiguity**  **(rocky scrub)** | **Habitat diversity** |
| --- | --- | --- | --- | --- | --- | --- | --- | --- | --- |
| **ALAN** | -0.27  (0.07) |  |  |  |  |  |  |  |  |
| **Predation pressure** | -0.26  (0.08) | 0.27  (0.08) |  |  |  |  |  |  |  |
| **Distance from city center** | 0.14  (0.37) | **-0.72**  **(<0.01)** | -0.22  (0.15) |  |  |  |  |  |  |
| **Proportion of built-up** | -0.22  (0.16) | **0.74**  **(<0.01)** | 0.27  (0.08) | **-0.59**  **(<0.01)** |  |  |  |  |  |
| **Proportion of rocky-scrub** | 0.17  (0.28) | 0.04  (0.81) | -0.08  (0.62) | 0.07  (0.63) | -0.1  (0.54) |  |  |  |  |
| **Rate of change (rocky scrub)** | -0.21  (0.17) | 0.18  (0.25) | 0.06  (0.71) | -0.15  (0.33) | 0.08  (0.59) | **0.36**  **(0.02)** |  |  |  |
| **Habitat contiguity**  **(rocky scrub)** | 0.21  (0.17) | -0.05  (0.74) | -0.14  (0.38) | 0.11  (0.48) | -0.15  (0.33) | **0.85**  **(<0.01)** | 0.23  (0.14) |  |  |
| **Habitat diversity** | -0.12  (0.45) | -0.18  (0.24) | -0.01  (0.93) | -0.05  (0.75) | -0.26  (0.09) | 0.18  (0.24) | 0.26  (0.09) | 0.14  (0.36) |  |
| **Proportion of cropland** | **0.54**  **(<0.001)** | **-0.45**  **(<0.001)** | -0.13  (0.40) | 0.21  (0.18) | **-0.41**  **(0.01)** | -0.09  (0.54) | **-0.33**  **(0.03)** | 0.03  (0.86) | 0.24  (0.12) |

**Supplementary Table 2.** Generalised additive models (GAM) and linear models (LM) to predict the relationship between the land use land cover types and the distance from the city centre. Separate models were run for each of the response variables. Approximate significance of the smooth terms are indicated by the smooth p-value. A significant p-value in GAM indicates that a horizontal line cannot be drawn within the 95% confidence interval of the model. Deviance explained (in %) is equivalent to pseudo R^2^ and shows how well the predictor explains the variation in the response variable. The minimised Generalised Cross-Validation (GCV) score indicates degree of model fit, such that smaller values of GCV are better fitting models. For example, GAM is a better model than LM for explaining how proportion of built-up changes with distance from the city centre.

| ***Predictor*** | ***Response*** | **GAM** | | | **LM** | | |
| --- | --- | --- | --- | --- | --- | --- | --- |
|  |  | ***smooth***  ***p value*** | ***Deviance explained*** | ***GCV*** | ***coeff***  ***p value*** | ***Deviance explained*** | ***GCV*** |
| **Distance from the city centre** | **Proportion of Built-up** | <0.01 | 70.4% | 0.019 | <0.01 | 32.7% | 0.041 |
|  | Proportion of cropland | <0.01 | 11.6% | 0.079 | <0.01 | 6.56% | 0.082 |
|  | Proportion of plantation | <0.01 | 7.33% | 0.074 | 0.40 | 0.35% | 0.078 |
|  | Proportion of forest | <0.01 | 12% | 0.047 | 0.047 | 1.98% | 0.049 |
|  | Proportion of water | 0.386 | 1.87% | 0.0086 | 0.861 | 0.02% | 0.0087 |
|  | Proportion of rocky scrub | 0.0219 | 2.62% | 0.013 | 0.0219 | 2.62% | 0.013 |

**Supplementary Table 3.** Generalised additive models (GAM) and linear models (LM) to predict the relationship between environmental variables and distance from the city centre and proportion of built-up as separate predictors. Approximate significance of the smooth terms are indicated by the smooth p-value. A significant p-value in GAM indicates that a horizontal line cannot be drawn within the 95% confidence interval of the model. Deviance explained (in %) is equivalent to pseudo R^2^ and shows how well the predictor explains the variation in the response variable. The minimised Generalised Cross-Validation (GCV) score indicates degree of model fit, such that smaller values of GCV are better fitting models. For example, GAM is a better model than LM for explaining how ALAN changes with distance from the city centre.

| ***Predictor*** | ***Response*** | **GAM** | | | **LM** | | |
| --- | --- | --- | --- | --- | --- | --- | --- |
|  |  | ***smooth***  ***p value*** | ***Deviance***  ***explained*** | ***GCV*** | ***coeff***  ***p value*** | ***Deviance***  ***explained*** | ***GCV*** |
| **Distance from the city centre** | Maximum LST (celsius) | <0.01 | 12% | 2.59 | 0.23 | 0.72% | 2.87 |
|  | ALAN | <0.01 | 64.60% | 124.82 | <0.01 | 50.80% | 163.38 |
|  | Avian predation pressure | <0.01 | 11% | 28.92 | <0.01 | 8.25% | 28.94 |
|  | Habitat contiguity (rocky scrub) | 0.01 | 3.16% | 639.57 | 0.01 | 3.16% | 639.57 |
|  | Habitat diversity | <0.01 | 20.50% | 0.03 | 0.37 | 0.40% | 0.04 |
|  | Rate of change (rocky scrub) | 0.19 | 2.93% | 2.31 | 0.66 | 0.10% | 2.35 |
|  | | | | | | | |
| **Proportion of built-up** | Maximum LST (celsius) | <0.01 | 6.38% | 2.71 | <0.01 | 6.38% | 2.71 |
|  | ALAN | <0.01 | 65% | 117.77 | <0.01 | 61.70% | 127.29 |
|  | Avian predation pressure | 0.24 | 1.90% | 31.16 | 0.20 | 0.83% | 31.29 |
|  | Habitat contiguity (rocky scrub) | 0.01 | 6.54% | 628.41 | <0.01 | 3.96% | 634.32 |
|  | Habitat diversity | <0.01 | 29.40% | 0.03 | <0.01 | 7.49% | 0.04 |
|  | Rate of change (rocky scrub) | 0.29 | 0.57% | 2.34 | 0.29 | 0.57% | 2.34 |

**Supplementary Figure 1.** Patterns of change in environmental variables as a function of the proportion of built-up. Shown are the non-linear relationships (solid coloured lines) of (a) LST, (b) ALAN (c) Avian predation pressure, (d) Habitat contiguity for the rocky-scrub landcover (e) Habitat diversity, and (f) Rate of change for the rocky-scrub landcover. Gray shaded region indicates the confidence intervals along the trendline.


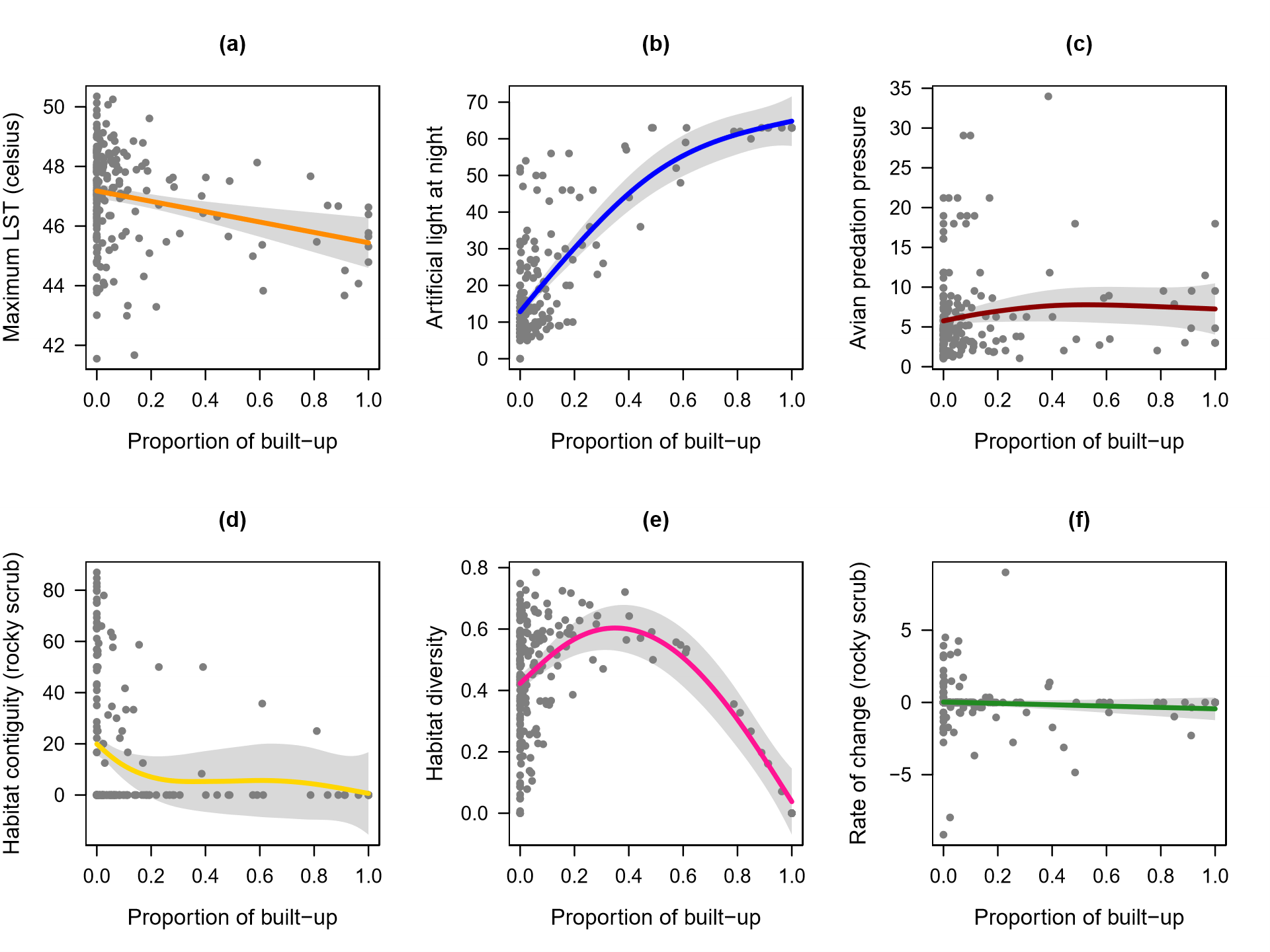

Supplement: Supplementary Material — The Supplementary Material for this article can be found online at: https://www.frontiersin.org/articles/10.3389/fcosc.2022.839836/full#supplementary-material [file EMS194938-supplement-Supplementary_Material.docx]
